# Workforce problems at rural public health-centres in India: a WISN retrospective analysis and national-level modelling study

**DOI:** 10.1186/s12960-021-00687-9

**Published:** 2022-01-28

**Authors:** Aatmika Nair, Yash Jawale, Sweta R. Dubey, Surabhi Dharmadhikari, Siddhesh Zadey

**Affiliations:** 1 Association for Socially Applicable Research (ASAR), Pune, 411007 Maharashtra India; 2 Rajarshi Chhatrapati Shahu Maharaj Government Medical College and CPR Hospital, Kolhapur, 416002 Maharashtra India; 3 Department of Bionanoscience, Kavli Institute of Nanoscience, Delft University of Technology, Delft, The Netherlands; 4 Duke Global Health Institute, Duke University, Durham, NC 27710 USA; 5 Department of Surgery, Duke University School of Medicine, Durham, NC 27710 USA

**Keywords:** Human resources for health, Workforce shortage, India, Rural health, Specialist doctors, WISN

## Abstract

**Background:**

Rural India has a severe shortage of human resources for health (HRH). The National Rural Health Mission (NRHM) deploys HRH in the rural public health system to tackle shortages. Sanctioning under NRHM does not account for workload resulting in inadequate and inequitable HRH allocation. The Workforce Indicators of Staffing Needs (WISN) approach can identify shortages and inform appropriate sanctioning norms. India currently lacks nationally relevant WISN estimates. We used existing data and modelling techniques to synthesize such estimates.

**Methods:**

We conducted a retrospective analysis of existing survey data for 93 facilities from 5 states over 8 years to create WISN calculations for HRH cadres at primary and community health centres (PHCs and CHCs) in rural areas. We modelled nationally representative average WISN-based requirements for specialist doctors at CHCs, general doctors and nurses at PHCs and CHCs. For 2019, we calculated national and state-level overall and per-centre WISN differences and ratios to depict shortage and workload pressure. We checked correlations between WISN ratios for cadres at a given centre-type to assess joint workload pressure. We evaluated the gaps between WISN-based requirements and sanctioned posts to investigate suboptimal sanctioning through concordance analysis and difference comparisons.

**Results:**

In 2019, at the national-level, WISN differences depicted workforce shortages for all considered HRH cadres. WISN ratios showed that nurses at PHCs and CHCs, and all specialist doctors at CHCs had very high workload pressure. States with more workload on PHC-doctors also had more workload on PHC-nurses depicting an augmenting or compounding effect on workload pressure across cadres. A similar result was seen for CHC-specialist pairs—physicians and surgeons, physicians and paediatricians, and paediatricians and obstetricians–gynaecologists. We found poor concordance between current sanctioning norms and WISN-based requirements with all cadres facing under-sanctioning. We also present across-state variations in workforce problems, workload pressure and sanctioning problems.

**Conclusion:**

We demonstrate the use of WISN calculations based on available data and modelling techniques for national-level estimation. Our findings suggest prioritising nurses and specialists in the rural public health system and updating the existing sanctioning norms based on workload assessments. Workload-based rural HRH deployment can ensure adequate availability and optimal distribution.

**Supplementary Information:**

The online version contains supplementary material available at 10.1186/s12960-021-00687-9.

## Background

Human resources for health (HRH) are critical to thriving health systems [1] with adequate levels and equitable distribution necessary for optimal health service delivery. India is one of the 57 countries with a critical HRH shortage [2]. The national density of doctors, nurses, and midwives was found to be 20.6 per 10 000 people [3] compared to the World Health Organization (WHO) recommendation of 44.5 [4]. It is noteworthy that the current health workers density is a significant improvement compared to the estimated 13.6 per 10 000 in 2005 [5]. Although, the concentration of HRH is inequitable across various states [6]. There are significant urban–rural differences in HRH with urban areas having four times greater doctor density than rural areas [5]. Therefore, India took up HRH expansion as a sustainable development goal (SDG)-2030 target indicator for achieving quality healthcare [7].

To enhance health services and improve HRH access in rural India, the Ministry of Health and Family Welfare (MoHFW) launched the National Rural Health Mission (NRHM) in 2005. Ten years into its implementation, HRH shortage still persists in rural areas [8]. Within NRHM, HRH sanctioning is based on the Indian Public Health Standards (IPHS) that proposes a three-tier health centre system based on underlying population densities and fixed catchment areas and provides the benchmarks for essential and desired health services delivery. For instance, a typical primary health centre (PHC) should have 1 MBBS doctor and 3 nurses to serve 30 000 people in non-hilly areas [9]. Health workforce sanctioning based on demand for health services and morbidity data of various geographical regions has been increasingly advocated in recent years [10, 11]. Against the backdrop of IPHS norms, Rural Health Statistics (RHS) 2014–15 reported high percentages of vacancy (deficit w.r.t. sanctioned positions) and shortfall (deficit w.r.t. required positions) owing to unavailability and suboptimal sanctioning in the rural public sector [9, 12, 13]. Therefore, there is a need for sanctioning based on empirical assessment of health workforce requirements according to demand.

WHO’s Workload indicators of staffing needs (WISN) tool dictates workload-based HRH allocation. The WISN method calculates the absolute (difference) and relative (ratio) facility-specific surplus or deficit for an HRH cadre. Previously, small scale WISN-based assessments have been conducted in India focusing on nurses working in maternity ward [14], emergency operation theatre [15], infection control [16] and emergency department [17] in tertiary care centres, nurses at rural hospitals [10] and doctors at PHCs [18, 19], CHCs, and SCs [19] (see summary in Additional file 1). Other countries such as Namibia have demonstrated the utility of nationally representative WISN study to evaluate the HRH inadequacies and optimise staffing needs across the country [20]. Currently, India lacks such a nationally applicable assessment.

To fill the gap, we focused on specialist doctors—physicians, surgeons, obstetricians and gynaecologists (OBGYNs), and paediatricians, general doctors (General Duties Medical Officers—GDMOs) and nurses at community health centres (CHCs), and doctors (MBBS Medical Officers—MOs) and nurses at PHCs in rural areas. We aimed to—(a) synthesise nationally relevant WISN-based requirement thresholds for these cadres using retrospective analysis of existing facility data and modelling techniques, (b) calculate national and state-level WISN differences and ratios to depict workforce problems and workload pressure for 2019 and (c) compare WISN-based requirements with existing HRH sanctioning to investigate sub-optimality at national and state levels for 2019. Our findings can inform national-level HRH policymaking and planning in the Indian rural public health system.

## Methods

### Data sources and variables

We extracted data for facility-level workload (health service and other activities) calculations from the Access, Bottlenecks, Costs, and Equity (ABCE) project surveys that collected data for the fiscal years 2008–09 to 2012–13 in Madhya Pradesh (MP) [21], 2010–11 to 2014–15 in Gujarat (GJ) [22], 2009–10 to 2013–14 in Odisha (OD) [23], 2007–08 to 2011–12 in Tamil Nadu (TN) [24], and 2007–08 to 2011–12 in Andhra Pradesh and Telangana (AP&TG) [25] at rural PHCs and CHCs. The ABCE project used stratified random-sampling to create nationally representative facility data sets. Facilities in rural and semi/peri-urban localities from the survey were taken as rural. We focused on 8 centre-cadre combinations in rural areas—PHC-nurses, PHC-doctors, CHC-nurses, CHC-GDMOs, CHC-physicians, CHC-surgeons, CHC-OBGYNs, and CHC-paediatricians. These cadres have specific activities that they perform at PHCs and CHCs according to IPHS (Additional file 2). Cadre-specific workload components extracted from ABCE were segregated into health service activities (HSA) (e.g., outpatient visits, inpatient admissions, surgeries, deliveries, etc.), support, and additional activities (e.g., patient review meetings, outreach services, administrative meetings) performed by all or select staff members (Additional file 3A–C). The patient numbers depicted the total annual workload of a particular service provided at a healthcare centre. We also extracted facility-level loads for support and additional services (Additional file 3A–C).

For activity standards (time required to perform the activity), we referred previously conducted WISN studies in India (see Additional file 1 for study details and Additional file 3A–C for variables) followed by WHO–WISN Methods Guide [26]. Activity standards were collected for the HSA included for doctor and nurse cadres at PHCs and CHCs. The standards were converted to common units (Additional file 3A–C).

To project WISN estimates at state (i.e., states and union territories) and national levels, we used cadrewise data on ‘in-position’ (actual staff present) and ‘sanctioned’ posts (under NRHM based on IPHS norms defined as authorized or approved positions) from RHS 2019 [27]. The numbers of functional rural PHCs and CHCs were also extracted. States with missing or incomplete data were excluded from the analysis (Additional file 4).

### WISN calculations for individual health centre facilities

We calculated annual available working time (AWT) in hours for each cadre according to

1 $${\text{AWT}} = \left[ {A - \left( {B + C + D + E} \right)} \right] \times F$$

where *A*, *B*, *C*, *D* and *E* are the numbers of working days in a year, annual leaves, sick leaves, public holidays and other leaves, respectively. F is the number of working hours per day. Values for leaves were taken from an existing WISN Indian study [10].

Standard workload represents the possible volume of HSA conducted by a health worker in a year. It was calculated by dividing AWT by the respective service activity standards. The annual workload was the actual number of patients seeking care under respective health services in that year. The required number of health workers for HSA was obtained by adding the ratios of annual workload to the standard workload for each health service.

Category allowance standard (CAS) expressed as percentage AWT spent, represents the activity standard for the given support activity of all staff members of a cadre. We used facility-reported actual working times and time standards from other sources (Additional file 3B). Total CAS percentage was the sum of individual CAS. Category allowance factor (CAF) is the multiplier that gives the required number of staff for health service and support activities. It was calculated as

2 $${\text{CAF}} = \frac{1}{{1 - \frac{{\text{Total CAS percentage}}}{100}}}$$

Individual allowance standard (IAS) represents the activity standard for a given additional activity of select staff members. IAS was the product of the time required to perform given additional activity and the number of staff members involved in the activity. We used the maximum value of actual working times reported among facilities. Total IAS was the sum of individual IAS. Individual allowance factor (IAF) is the staff required to cover additional activities and was calculated as

3 $${\text{IAF = }}\frac{{\text{Total IAS}}}{{{\text{AWT}}}}$$

The WISN-based required number of staff of an HRH cadre at a health centre facility was calculated as

4 $${\text{WISN = }}\left( {{\text{HSA }} \times {\text{ CAF}}} \right){\text{ + IAF }}$$

The raw values for facility-specific WISN-based requirements for cadres were rounded to integers as per WISN user’s manual [28].

We excluded facilities that resulted in null values (WISN = 0). Given that IAF forms a significant proportion for nurses’ workload, data points with null values for this component were excluded for nurses at PHCs and CHCs. We assumed a standard workweek to be 48 h (8 h × 6 days) and considered that some facilities might operate on a partial basis. Facilities with < 24 average working hours per week that did not seem to reach half-the-standard workweek were excluded. Hence, facilitywise WISN values were calculated for 8 centre-cadre combinations mentioned above.

### Modelling nationally representative average for WISN-based requirements

To explore data heterogeneity, facility-specific raw (unrounded) WISN values for all cadres were assessed for across-state differences using non-parametric Kruskal–Wallis one-way ANOVA (analysis of variance). We used raw values for better ANOVA model fit as count data generated by WISN rounding scheme created saturation issues. Non-parametric tests were chosen due to observed skewness in data. To create WISN-based cadre requirement values that could be suitably used for national-level planning, generalised estimation equations (GEE) [29]. GEE estimates population-averaged responses and is robust to covariance mis-specification. Since we used data collected over years from facilities clustered within states to create nationally relevant WISN-based requirement thresholds, we used GEE to control the effects of these variables, i.e., estimates averaged over states and years. Here, the log-link Poisson model permitted the use of rounded WISN values as count outcome with state and year as categorical predictors. For each centre-cadre combination (e.g., PHC-doctors), three models with different working error correlation structures (independence, exchangeable, and auto-regressive order-1) were run. The model with the lowest quasi information criterion (QIC) value was chosen to represent the data best. Predicted marginal means and 95% asymptotic confidence intervals for the best-fit model gave WISN-based requirement values to represent average per-centre estimates for India, accounting for the influence of individual states and years:

5 $${\text{log}}\left( {{\text{WISN}}_{ij} } \right) = \beta_{0} + \beta_{1} {\text{State}} + \beta_{2} {\text{Year }}$$

where *i* = health-centre facility ID, *j* = measurement instance.

### National and state-level WISN projections

WISN ratios, per-centre and overall WISN differences were calculated for states and all India as follows:

6 $${\text{WISN ratio}} = \frac{P}{{{\text{WISN}} \times N}}$$

7 $${\text{WISN difference}} \left( {\text{per - centre}} \right) = \left( \frac{P}{N} \right) - {\text{WISN}}$$

8 $${\text{WISN difference}} \left( {{\text{overall}}} \right) = P - \left( {{\text{WISN}} \times N} \right)$$

where ‘WISN’ stands for the nationally representative modelled average WISN-based requirement threshold for a centre-cadre combination, ‘*P*’ stands for the actual total number of staff of the cadre present at the given centre (PHC and CHC) at state and national levels, and ‘*N*’ represents the number of functional centres of the type (PHCs and CHCs) at the state and national levels from RHS-2019. The interpretation of the values was as per the WISN user’s manual [28]. WISN difference depicted workforce problem, categorised as balance, surplus and shortage based on values = 0,  > 0, and < 0, respectively. WISN ratio implied workload pressure, with values = 1 and > 1 indicating normal pressure and no pressure, respectively. For ratios < 1, we created arbitrary categories for WISN ratio for the current study as follows:

0–0.25 = very high, 0.25–0.50 = high, 0.50–0.75 = medium, and 0.75–1 = low. The WISN ratios are categorized into 6 groups (0–0.25, 0.25–0.50, 0.50–0.75, 0.75–1, 1, > 1) and are interpreted together with WISN differences to determine the workload pressure.

To assess the association of workload pressure across states for HRH cadres at a given centre type, we calculated nonparametric Spearman’s rank correlations (ρ). We chose Spearman’s correlations as they are robust to linearity and normality assumptions and biases due to outliers and small samples [30]. For PHCs, a bivariate correlation was calculated between doctors and nurses across states. For CHCs, we calculated partial correlations among the 6 HRH cadres to determine workload pressure co-occurrence between specific cadre pairs while controlling for other interactions.

### Comparison of WISN-based requirement with current sanctioning

Two analyses were conducted to investigate suboptimal sanctioning. First, we calculated:

9 $${\text{Sanctioning difference}} \left( {\text{per - centre}} \right) = \left( \frac{S}{N} \right) - {\text{WISN}}$$

10 $${\text{Sanctioning difference }}\left( {{\text{overall}}} \right) = S - \left( {{\text{WISN}} \times N} \right)$$

where ‘WISN’ and ‘*N*’ stand for values as described above, while ‘*S*’ stands for the total number of sanctioned posts of a cadre at the given centre type (PHC and CHC) at the state and national levels from RHS-2019. Sanctioning differences depict HRH misallocation with values > 0 indicating over-sanctioning, < 0 indicating under-sanctioning, and = 0 indicating optimal sanctioning. Second, we checked the concordance (i.e., agreement) between the sanctioned posts under the current norm (*S*) and WISN-based requirements (WISN*N, as given above) across states using Lin’s concordance correlation coefficient (R_C_) [31]. Coefficient values of −1, 0, and + 1 depict perfect disagreement, no agreement, and perfect agreement, respectively. Values < 0.90 depict poor agreement [32]. We also calculated the bias correction factor that measures the deviation from 45^°^ line (perfect concordance), with 1 showing no deviation.

### General statistical and packages details

Statistical significance for hypothesis tests (ANOVA and correlations) was set at the conventional threshold of 0.05, i.e., *p* values < 0.05, were considered significant. Analyses were conducted in open-source R (Version 4.0.2) [33] and R-Studio (Version 1.3.1056) (https://rstudio.com/) using validated packages [34–42]. We provide the analysis scripts (Additional file 5A–D), generated data (Additional file 6A–B) and data dictionary for RHS-based calculations (Additional file 6C). These files can also be viewed on https://github.com/asarforindia/RHS-WISN.

## Results

### Facility-level WISN calculations

Data from 93 facilities across 5 states and 8 years were used after excluding discrepant data points. State-year cross-tabulations for centre-cadre combinations along with missing data are presented in Table 1. The rounded WISN values (mean ± standard deviation) for nurses (no. of data points: *n* = 98) and doctors (*n* = 170) at PHCs were 11 ± 5 and 2 ± 2. For nurses (*n* = 79) and GDMOs (*n* = 72) at CHCs, average WISN were 63 ± 25 and 4 ± 3, respectively. The WISN was 2 ± 1 for physicians (*n* = 178), surgeons (*n* = 133), OBGYNs (*n* = 110), and paediatricians (*n* = 209) at CHCs. Statistically significant (*p* < 0.001) across-state differences were seen for all centre-cadres (Additional file 7: Fig. S1A–B). Hence, averaging out state variability was crucial for creating nationally-relevant WISN-based thresholds.

**Table 1:** Data point counts and missing data points for facilities from ABCE

PHC-nurses	AP & TG	GJ	MP	OD	TN
2007	16	–	–	–	–
2008	15	–	–	–	–
2009	18	–	–	–	–
2010	17	2	–	–	–
2011	22	2	–	–	–
2012	–	2	–	–	–
2013	–	2	–	–	–
2014	–	2	–	–	–
PHC-doctors	AP & TG	GJ	MP	OD	TN
2007	16	–	–	–	3
2008	15	–	–	–	3
2009	18	–	–	4	3
2010	17	8	-	4	3
2011	22	9	–	4	3
2012	–	10	–	4	-
2013	–	11	–	4	-
2014	–	9	–	–	–
CHC-nurses	AP & TG	GJ	MP	OD	TN
2007	3	–	–	–	–
2008	2	–	1	–	–
2009	3	–	1	10	–
2010	4	2	1	10	–
2011	2	2	1	10	–
2012	–	2	1	10	–
2013	–	2	–	10	–
2014	–	2	–	–	–
CHC-GDMOs	AP & TG	GJ	MP	OD	TN
2007	8	–	–	–	–
2008	7	–	1	–	–
2009	8	–	1	3	–
2010	9	2	1	3	–
2011	7	2	1	3	–
2012	–	3	1	3	–
2013	–	3	–	3	–
2014	–	3	–	–	–
CHC-physicians	AP & TG	GJ	MP	OD	TN
2007	12	–	–	–	–
2008	12	–	2	–	–
2009	12	–	2	18	–
2010	12	3	2	18	–
2011	12	3	2	18	–
2012	–	4	2	18	–
2013	–	4	-	18	–
2014	–	4	–	–	–
CHC-surgeons	AP & TG	GJ	MP	OD	TN
2007	12	–	–	–	–
2008	12	–	5	–	–
2009	12	–	5	6	–
2010	12	3	5	6	–
2011	12	3	5	7	–
2012	–	3	5	7	–
2013	–	3	-	7	–
2014	–	3	–	–	–
CHC-OBGYNs	AP & TG	GJ	MP	OD	TN
2007	3	–	–	–	–
2008	2	–	3	–	–
2009	3	–	3	12	–
2010	4	4	3	11	–
2011	4	4	3	11	1
2012	–	4	3	11	-
2013	–	5	–	11	–
2014	–	5	–	–	–
CHC-paediatricians	AP & TG	GJ	MP	OD	TN
2007	10	–	–	–	–
2008	10	–	9	–	–
2009	10	–	10	18	–
2010	10	3	10	18	1
2011	9	3	10	18	2
2012	–	4	10	18	–
2013	–	4	–	18	–
2014	–	4	–	–	–

‘–’ depicts no data available. ABCE, Access, Bottlenecks, Costs, Equity; PHC, Primary Health Centre; CHC, Community Health Centre; GDMO, General Duties Medical Officer; OBGYNs, Obstetricians and Gynaecologists; AP & TG, Andra Pradesh and Telangana; OD, Odisha; MP, Madhya Pradesh; TN, Tamil Nadu

### Modelled WISN-based requirements for India

Centre-cadre specific WISN-based values (raw and rounded) estimated using GEE against the current norms from revised IPHS guidelines are presented in Table 2. The estimates reflect the nationally-relevant average number of staff required per centre based on workload distribution. The modelled estimates agree with the unweighted means presented above for general (PHC-doctors and CHC-GDMOs) and specialist doctors (CHC-physicians, surgeons, OBGYNs, and paediatricians) partly due to the effect of WISN rounding on small values. However, these estimates vary from the unweighted averages for nurses at PHCs and CHCs depicting the model utility that accounts for across-state and over-years variability.

**Table 2:** Generalized estimating equations (GEE) results for cadre-specific WISN-based requirement values averaged-over states and years

Centre-cadre combination	Current norms according IPHS (Revised 2012)	*n*	Mean WISN-based need (rounded) [95% CI]	Mean WISN-based need (raw) [SE]	QIC	Working correlation matrix (GEE)
PHC-nurses	4	98	15 [13, 16]	14.867 [0.752]	−2877.731	Exchangeable
PHC-doctors	1	170	2 [2, 3]	1.834 [0.186]	258.998	Exchangeable
CHC-GDMOs	2	72	4 [3, 4]	3.328 [0.293]	−246.012	Auto-correlation
CHC-nurses	10	79	45 [42, 49]	45.145 [1.658]	−32252.368	Independence
CHC-physicians	1	178	2 [2]	1.579 [0.075]	158.300	Independence
CHC-surgeons	1	133	2 [2]	1.317 [0.115]	231.274	Auto-correlation
CHC-OBGYNs	1	110	2 [1, 2]	1.113 [0.058]	258.702	Auto-correlation
CHC-paediatricians	1	209	2 [2]	1.339 [0.073]	330.810	Independence

*n* is the number of data points. SE, standard error of mean; CI, confidence interval; QIC, quasi-information criterion; IPHS, Indian Public Health Standards

### National-level WISN differences and ratios

**Fig. 1:**
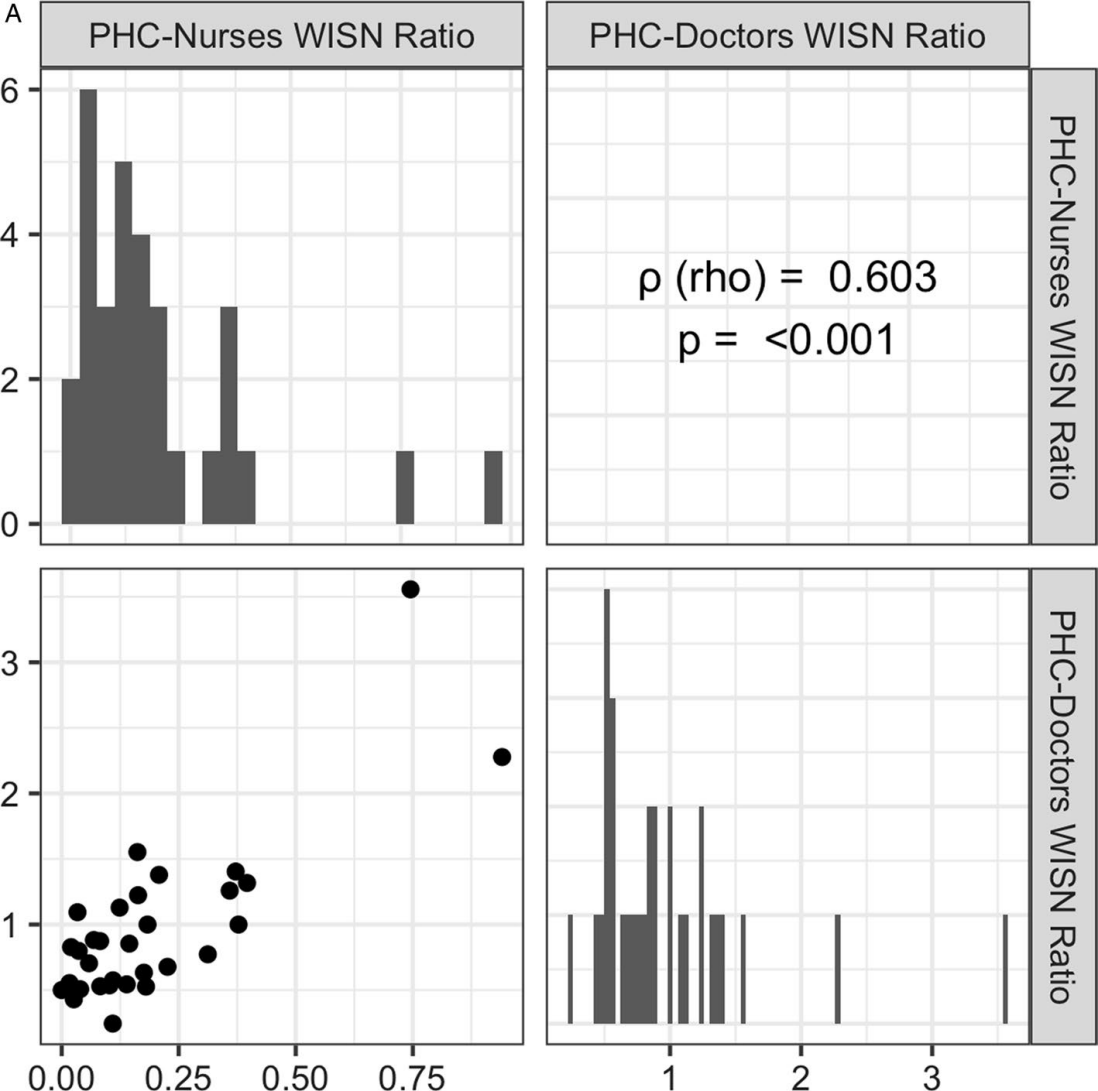

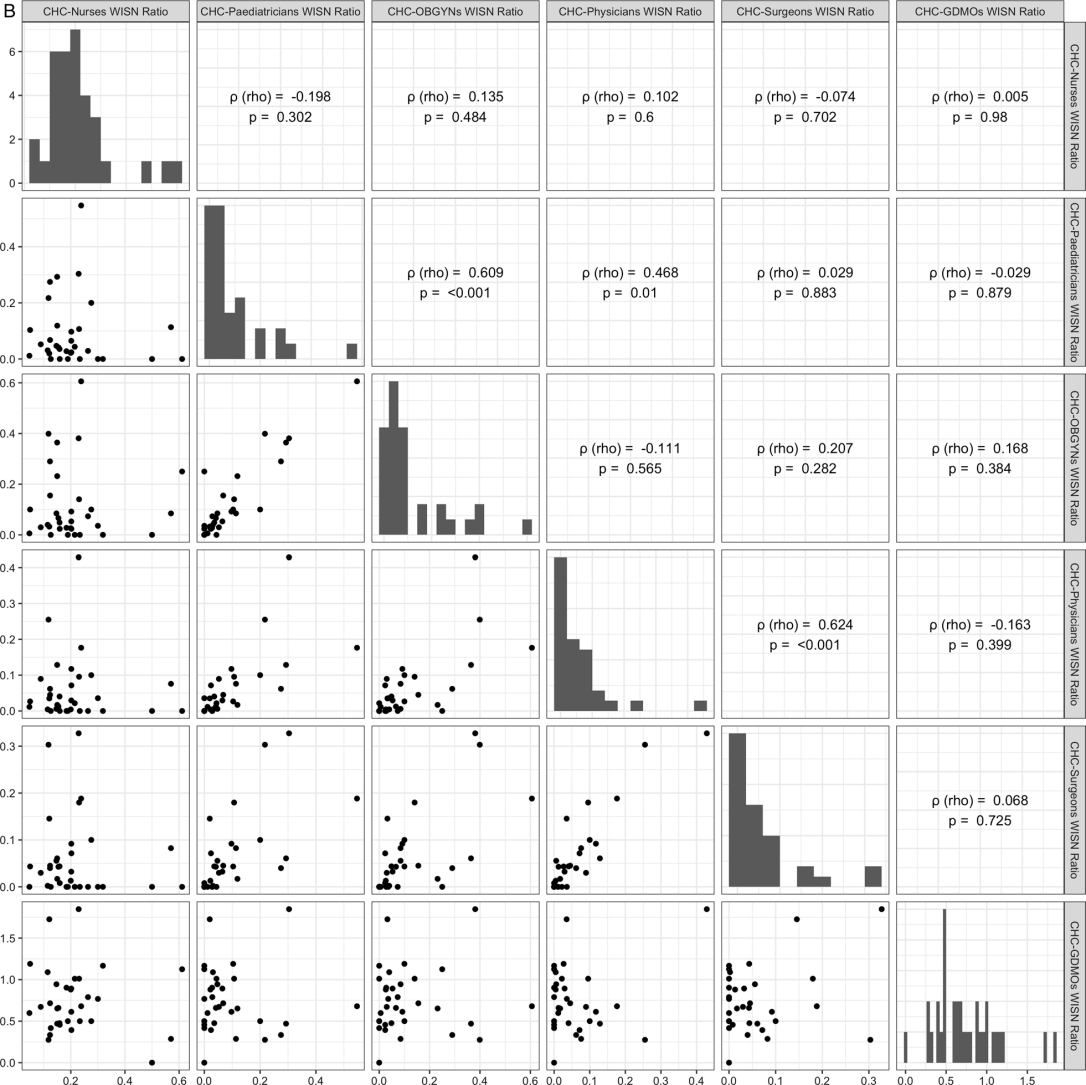
**A**, **B** Spearman’s rank correlations between state-level WISN ratios of HRH cadres at **A** PHCs and **B** CHCs. Rho (ρ) represents pairwise correlation in **A** and partial correlations in **B**

In 2019, at the national level, rural PHCs and CHCs faced acute workforce problems (Table 3). All HRH cadres had workforce shortages depicted by negative values for per-centre and overall WISN differences. WISN ratios showed that nurses at PHCs and CHCs, and all specialist doctors at CHCs had very high workload pressure. There was a significant positive correlation of large magnitude between WISN ratios for doctors and nurses at PHCs across 31 states (Fig. 1A). States with more workload on PHC-doctors also had more workload on PHC-nurses depicting an augmenting or compounding effect on workload pressure across cadres. Significant positive partial correlations were observed for three specialist pairs- physicians and surgeons, physicians and paediatricians, and paediatricians and OBGYNs after controlling for the correlations with all other cadres at CHCs across 33 states (Fig. 1B). Hence, states had a compounding workload pressure only for certain specialist doctor-pairs that often work closely together at CHCs.

**Table 3:** Workforce, workload and sanctioning problems of rural HRH at national-level, India

Centre-cadre combination	WISN difference per centre	WISN difference overall	Workforce problem	WISN ratio	Workload Pressure	Sanctioning difference per centre	Sanctioning difference overall	Sanctioning problem (compared to IPHS 2012 norms)
PHC-Nurses	−13.19	−219124	Shortage	0.121	Very high	−13.23	−219719	Under-sanctioning
PHC-Doctors	−0.21	−3427	Shortage	0.897	Low	−0.02	−402	Under-sanctioning
CHC-Nurses	−35.46	−189170	Shortage	0.212	Very high	−37.63	−200750	Under-sanctioning
CHC-GDMOs	−1.11	−5945	Shortage	0.721	Medium	−0.92	−4892	Under-sanctioning
CHC-Physicians	−1.87	−9987	Shortage	0.064	Very high	−1.47	−7845	Under-sanctioning
CHC-Surgeons	−1.86	−9902	Shortage	0.072	Very high	−1.40	−7469	Under-sanctioning
CHC-OBGYNs	−1.75	−9319	Shortage	0.127	Very high	−1.38	−7336	Under-sanctioning
CHC-Paediatricians	−1.80	−9591	Shortage	0.101	Very high	−1.39	−7433	Under-sanctioning

IPHS, Indian Public Health Standards

### State-level WISN differences and ratios

**Fig. 2:**
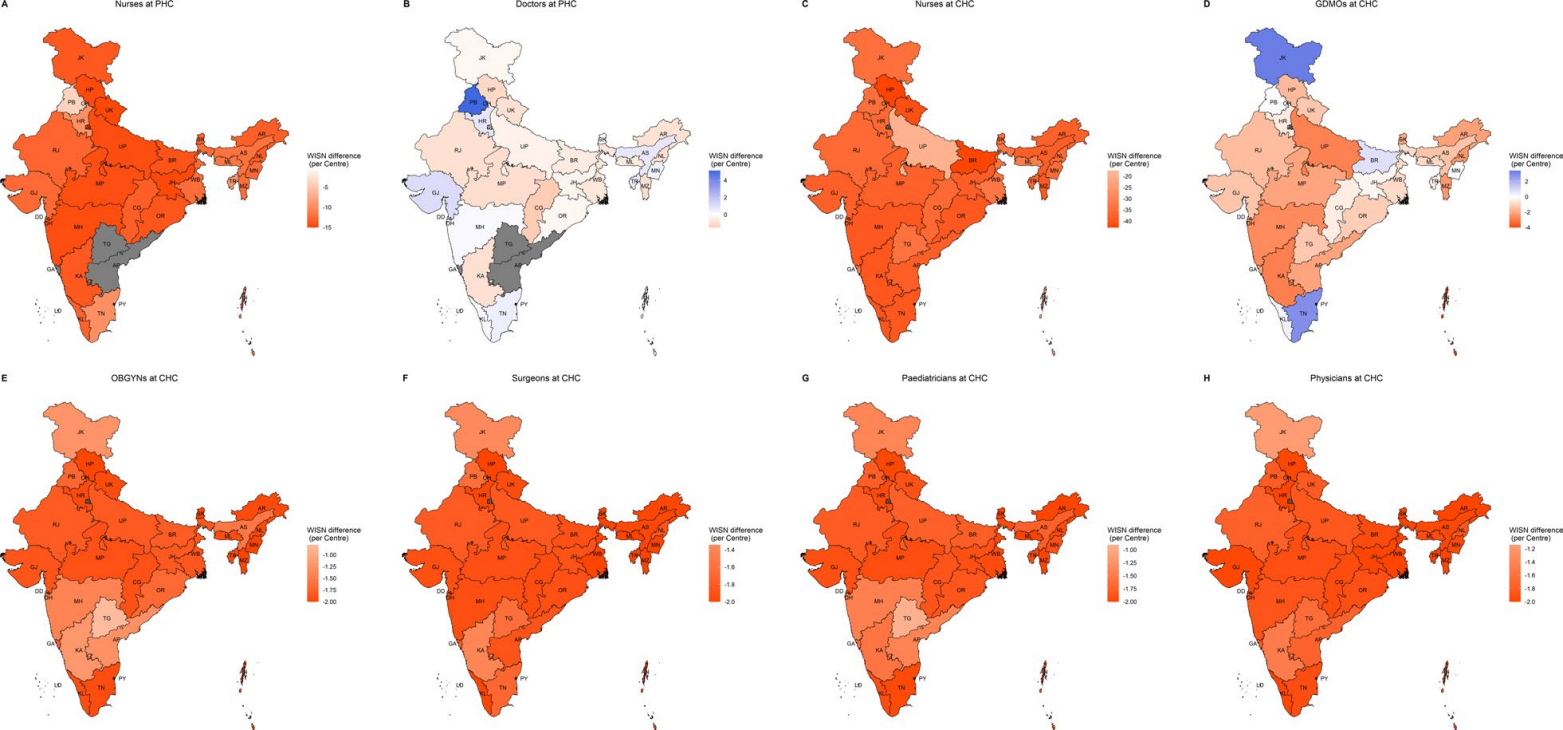
**A**–**H** Maps for per centre WISN differences for doctors and nurses at primary and community health centres (PHCs and CHCs). GDMOs = General Duty Medical Officers, OBGYNs = Obstetricians and Gynaecologists

**Fig. 3:**
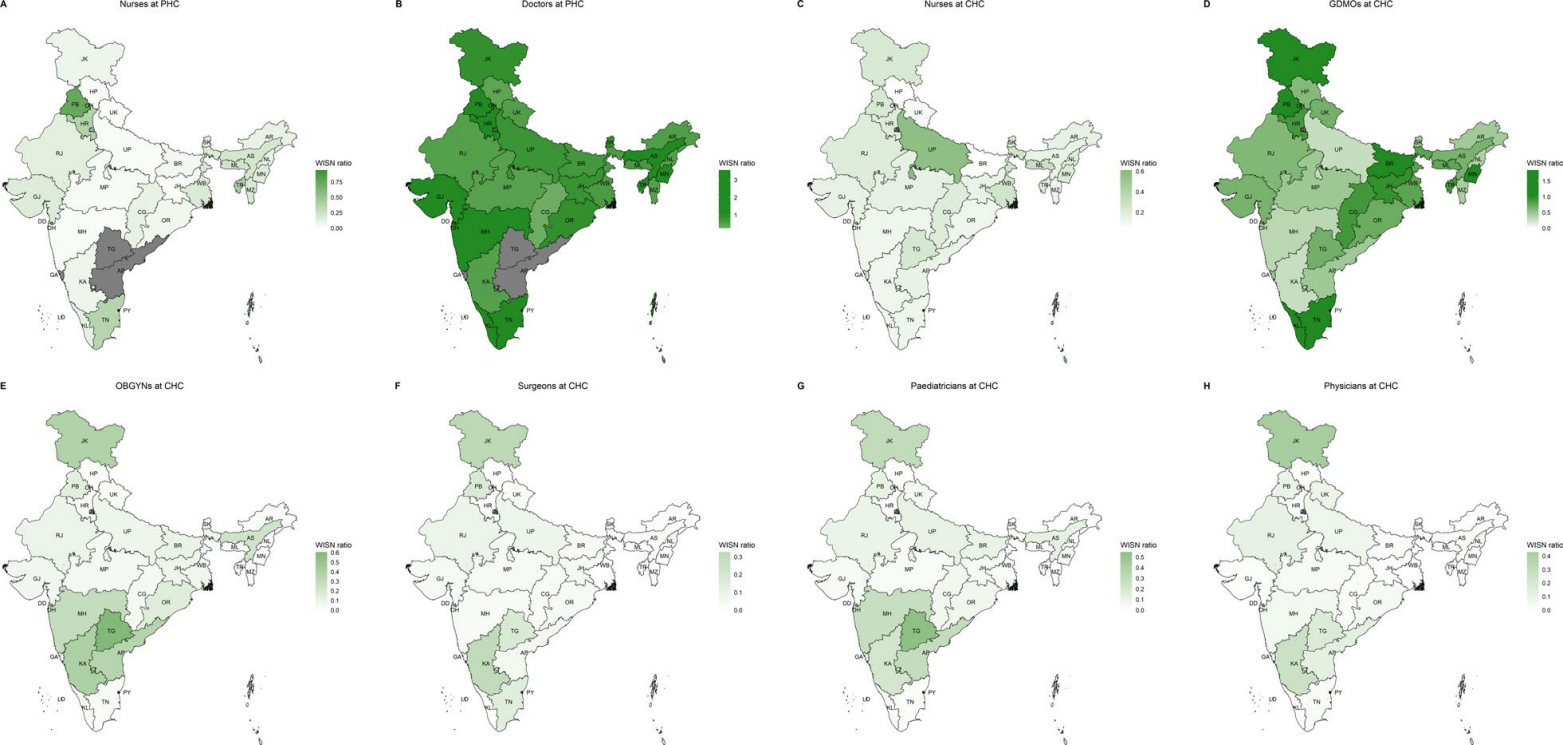
Maps for WISN ratios for doctors and nurses at primary and community health centres (PHCs and CHCs). GDMOs = General Duty Medical Officers, OBGYNs = Obstetricians and Gynaecologists

Across-state variability in per-centre WISN differences can be seen in Fig. 2A–H and Additional file 6B. All states had a shortage of nurses at PHCs (Fig. 2A) and CHCs (Fig. 2C), and all specialists at CHCs (Fig. 2E–H). Seven states had surplus doctors at PHCs, while others faced shortage (Fig. 2B). Four states had surplus GDMOs at CHCs, while others faced varying degrees of shortage (Fig. 2D). The results were qualitatively similar for state-level overall WISN differences (Additional file 8: Fig. S2A–H). Across-state variability in WISN ratios can be seen in Fig. 3A–H and Additional file 6B. PHC-doctors (Fig. 3B) mostly had no-to-medium workload pressure with Chhattisgarh, Delhi, and Himachal Pradesh as the only exceptions. Workload pressure on CHC-GDMOs (Fig. 3D) was very high for Andaman & Nicobar Islands, high for 11 other states, and no-to-medium for others. Nurses at PHCs and CHCs in most states had high or very high workload pressure with few exceptions. PHC-nurses in Puducherry and Punjab had low and medium pressure, respectively (Fig. 3A). CHC-nurses in Puducherry and Uttar Pradesh had medium pressure (Fig. 3C). All CHC-specialists had high and very high workload pressure in all states (Fig. 3E–H).

### Comparison of WISN-based requirement with current sanctioning

**Fig. 4:**
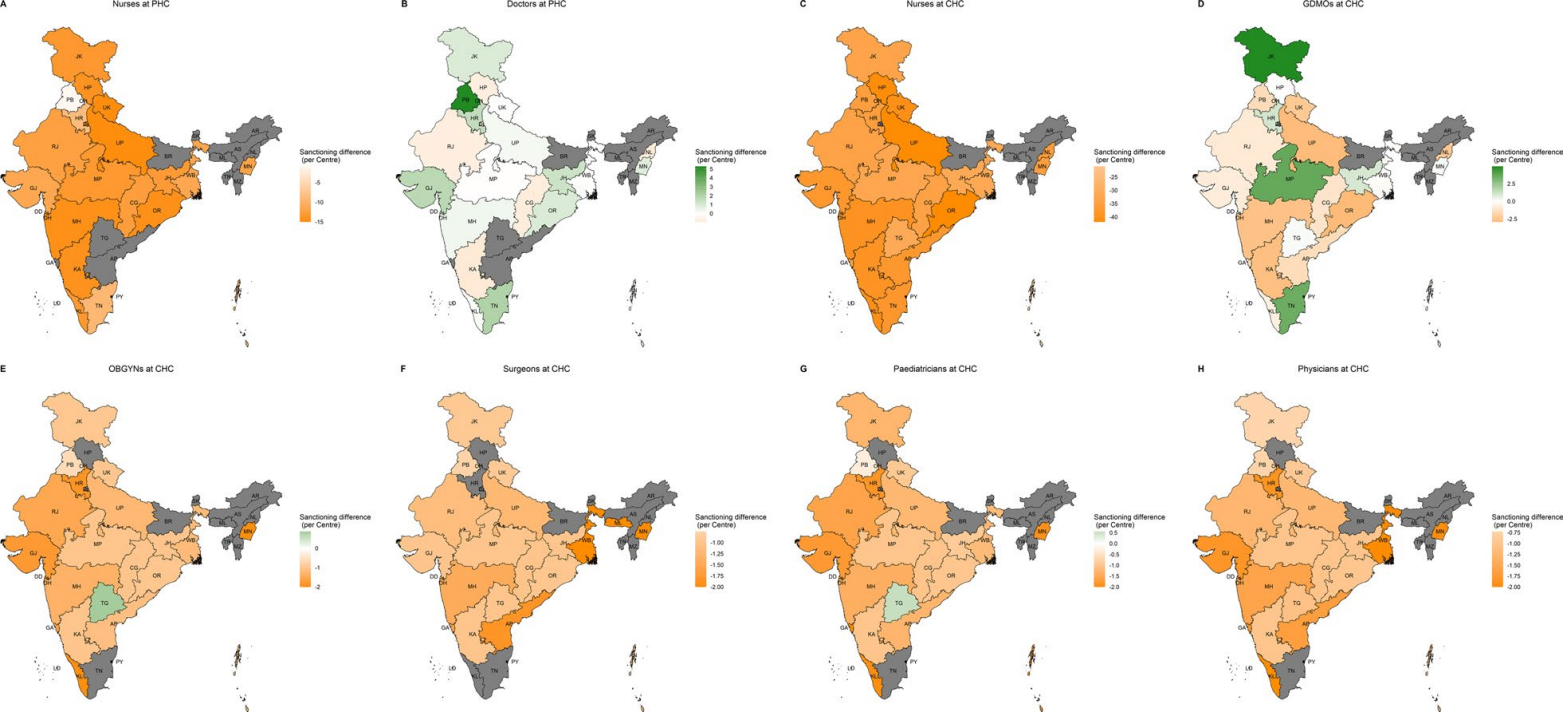
Maps for sanctioning differences for doctors and nurses at primary and community health centres (PHCs and CHCs). GDMOs = General Duty Medical Officers, OBGYNs = Obstetricians and Gynaecologists

All the centre-cadres suffered under-sanctioning at the national level given by < 0 overall and per-centre sanctioning differences with acute problems for nurses at PHCs and CHCs (Table 3). Across-state variability in per-centre sanctioning differences can be seen in Fig. 4A–H and Additional file 6B. Nurses at PHCs (Fig. 4A) and CHCs (Fig. 4C), as well as physicians (Fig. 4H) and surgeons (Fig. 4F) at CHCs, were under-sanctioned in all states. Except Telangana, OBGYNs (Fig. 4E) and paediatricians (Fig. 4G) at CHCs were under-sanctioned in all states. PHC-doctors (Fig. 4B) were under-sanctioned in 9 states and over-sanctioned in 13 others. CHC-GDMOs (Fig. 4D) were under-sanctioned in 17 states and over-sanctioned in 8 others. The results were qualitatively similar for state-level overall sanctioning differences (see Additional file 9: Fig. S3A–H).

We found poor concordance between current sanctioning and WISN-based state-level requirements for all centre-cadre combinations (*R*_C_ < 0.9) with the poorest agreement for PHC-nurses (Table 4).

**Table 4:** Concordance correlations between sanctioned and WISN-based required HRH across states

Centre-cadre combination	*N*	*R*_C_ [95% CI]	Bias correction factor
PHC-nurses	23	0.08 [0.01, 0.15]	0.15
PHC-doctors	24	0.85 [0.69, 0.93]	1
CHC-nurses	25	0.16 [0.07, 0.25]	0.21
CHC-GDMOs	25	0.63 [0.32, 0.82]	0.99
CHC-physicians	23	0.39 [0.22, 0.55]	0.49
CHC-surgeons	22	0.51 [0.33, 0.65]	0.56
CHC-OBGYNs	23	0.41 [0.24, 0.56]	0.49
CHC-paediatricians	23	0.4 [0.22, 0.55]	0.48

*N*, number of states; *R*_C_, Lin's Concordance Correlation Coefficient. Bias correction factor of 1 depicts no deviation from line of perfect concordance

## Discussion

To our knowledge, this is the first study to synthesise nationally-applicable WISN thresholds for nurses and doctors at PHCs and nurses, doctors and specialists at CHCs in rural India. Our retrospective analysis calculated WISN for 8 HRH cadres in 93 centres across 5 states. Based on these data, we modelled average WISN controlling for across-state and over-years differences to make the WISN-based requirement thresholds nationally-representative. Applying the modelled WISN thresholds to India, we found a shortage of nurses at PHCs and CHCs and specialist doctors at CHCs that suffered very high workload pressure. We found strong correlations between workload pressures on doctors and nurses at PHCs and for certain specialist cadre pairs at CHCs. For such pairs, states with a shortage of one cadre also face a shortage for another cadre at the given centre-level. The co-occurrence of shortages creates an augmenting or compounding effect on across-cadre workload pressure that can worsen the centre’s healthcare provision capacity. Through two analyses, we revealed that current sanctioning norms do not agree with WISN-based requirements for all HRH cadres, falling particularly short for nurses and specialist doctors. Based on these findings, Indian HRH policies should prioritise nurses and specialists in the rural public health system and update the existing sanctioning norms based on workload assessments. We attempted to demonstrate the utility of WISN calculations based on available data and modelling techniques for national-level policymaking.

Adaptations to WISN, in the absence of required data or presence of differing data, have been previously used to identify local problems and plan national-level HRH. For instance, a regional pilot’s success in Namibia to evaluate the staffing needs and workload distribution led to extending the findings to the national level [20]. Another study in Iran adapted WISN to include additional ‘uncommon’ activities to improve the precision of single-hospital-level optometrist requirements [43]. The adaptations and approximations made in the current analysis showcase how a retrospective assessment of existing data could be used for WISN calculations. While imperfect, such assessment is inexpensive and can generate insights for future data collection.

An Indian study used WISN to calculate the staff requirement for providing maternal and child services at SCs, PHCs, and CHCs in Ganjam district, Odisha [19]. The demand data calculations involved populations in the service area for each centre. Their findings for 18 centres in a single district, suggested the need for an increase of 43 doctors and 15 nurses, among other cadres. Our state-level results for Odisha suggest a shortage for doctors and nurses similar to the study. Still, we recommend a greater influx of nurses than general doctors at both PHC and CHC levels for the state. Another WISN study calculated the doctors required for Visceral Leishmaniasis active case detection at 4 PHCs in Bihar [18]. The study found a surplus of doctors at some PHCs while shortage at others pointing to misallocation. Our state estimate for Bihar that considers several health-service and other activities depicts an average shortage of 2 doctors per centre. The small magnitude and limited cadre focus of the past studies make their comparison with other large-scale analyses that contribute to national HRH planning difficult.

Several studies have recorded HRH shortage in the rural public sector (see [44] for review). A national-level assessment of RHS-2011 showed a shortfall of 2866 PHC-doctors with an indication for future rise [45]. Using the WISN approach, we found a shortage of 3427 PHC-doctors in 2019, agreeing with the expected rise. Another RHS-2015 evaluation showed Mizoram, Tamil Nadu, and Sikkim to have the highest shortage for specialists [8]. Contrarily, we found an acute shortage in Uttar Pradesh and Rajasthan based on overall WISN differences. The differences in findings can be attributed to different requirement thresholds and analysed RHS years. A cross-sectional study [46] of 13 CHCs in the Bharatpur district of Rajasthan suggested average per centre deficits of ~ 1 surgeon and paediatrician and ~ 4 nurses (staff nurses and midwives) using requirement norms from IPHS 2010 revised draft. Our findings for Rajasthan depict an average shortage of 2 surgeons and paediatricians and 36 nurses per centre. The large difference in nurse shortages could be due to the inclusion of midwives in [46]. Even so, these differences point to the utility of workload-based sanctioning in the rural public health system.

Unlike WISN, current IPHS guidelines define centre-contingent norms for HRH sanctioning that do not account for workload pressure on HRH cadres and differences in healthcare demands. Our findings depicted under-sanctioning across several states and showed limited concordance between WISN-based requirement and sanctioning, particularly for nurses and specialist doctors. There is an urgent need for NRHM’s programmatic scale-up for these cadres to meet rural India’s health demands.

## Limitations

The current study has the following limitations. First, the health service, support and additional activities are not exhaustive of all essential activities to be conducted at PHCs and CHCs under IPHS due to insufficient ABCE data. However, we ensured to include high-priority essential services (e.g., maternal and child health services and immunisation services) relevant for NRHM. Second, the use of approximations due to the lack of standards for certain activities could skew our WISN values. Third, in the absence of specific activity standards for some cadres and centres, we extrapolated the available standards to all centre-types and HRH cadres that could undermine essential differences. However, the average time taken for an activity by a particular cadre should be alike across similarly resourced centres. Fourth, we used a single estimate for leaves across states and facilities that could undermine local differences. Since all rural public health centres tend to operate under NRHM, the annual sum of leaves should be similar. Fifth, we assumed that WISN modelled averaging out differences among 5 states are nationally-representative. Additional data from other states could change these estimates. However, the facility-sampling frames used in the ABCE project were given to be nationally representative. Finally, we could not create WISN estimates for all states due to missing RHS data. Hence, future WISN studies should consider large nationally-representative facility samples focusing on multiple HRH cadres.

## Conclusions

Through WISN-based assessment, we attempted to determine the rural public health system’s workforce problems and inform national-level HRH planning in India. Our findings point towards the need for an evidence-based update of the current sanctioning norms. Deploying HRH in rural areas based on workload will ensure adequate availability and equitable distribution necessary for improving the overall quality of rural healthcare.

India currently lacks a dedicated HRH policy. Even then, sections of India’s National Health Policy (2017) focus heavily on doctors’ and nurses’ availability and quality in rural areas and recommend increasing HRH production and improving training [47]. Our findings make a case that the future NHPI recommendations for HRH deployment in the rural public sector could benefit from WISN assessments. India has selected SDG indicator 3.c.1 to achieve HRH density of 45 doctors, nurses and midwives per 10 000 people by 2030 [7]. Considering the urban–rural HRH differences, achieving the SDG target for rural India requires evidence-based HRH policy and planning and appropriate demand-based upscaling of specific cadres in the public health system.

## Supplementary Information

**Additional file 1: **Summary of WISN studies in India. Brief literature review of objectives and results of previous Indian WISN studies.

**Additional file 2:** Cadre-centre services as per IPHS’. Data on health service activities for cadre-centre combinations as per Indian Public Health Standards guidelines.

**Additional file 3: A** Health service activities (HSA) for cadre-centre combinations for 5 states in ABCE. Data on health service activities for cadre-centre combinations across 5 states from ABCE survey and other listed references. **B** Category allowance standards (CAS) for cadre-centre combinations for 5 states in ABCE. Data on support activities for cadre-centre combinations with category allowance standards from ABCE survey and other listed references. **C** Individual allowance standards (IAS) for cadre-centre combinations for 5 states in ABCE. Data on additional activities for cadre-centre combinations with individual allowance standards from ABCE survey and other listed references.

**Additional file 4:** Missing data in Rural Health Statistics (RHS). Missing data in the Rural Health Statistics 2019 with variable names and states given.

**Additional file 5: A** This R markdown contains code for WISN calculations conducted for ABCE facility survey data. The output file generated by the R code is used as input for Additional file 4B. **B** This R markdown contains code for multiple analyses and generates Tables 2 and 3, Additional file 5A, and figures in Additional file 6A–B among other interim outputs. It inputs data from Additional file 4A and provides input for Additional file 4C. **C** This R markdown contains code for multiple analyses and generates Figs. 2A–H, 3A–H, and 4A–H, Additional file 5B–C, figures in Additional files 7 and 8, and data for Table 1. It inputs some data from Additional file 4B and RHS source. **D** This R markdown contains code for some post analysis and generates Fig. 1A–B, Table 4 and extra figure related to Table 3. It inputs data from Additional file 4C.

**Additional file 6: A** Dataset for WISN calculations for Access, Bottlenecks, Costs, and Equity (ABCE) Project facilities. Data includes HSA, CAF, IAF, and WISN values calculated for rural PHC and CHC facilities in the ABCE surveys. Values were used further for modelling national-level WISN thresholds. **B** Dataset for national and state RHS-WISN calculations and interpretations. Data provides state and national-level numeric values of Sanctioned (from RHS), In-position (from RHS), WISN, WISN differences (overall and per centre), WISN ratios, and Sanctioning differences (overall and per centre), and categorical labels for corresponding workforce problems, workload pressure, and sanctioning problems for cadre-centre combinations. **C** Data dictionary for Dataset for national and state RHS-WISN calculations and interpretations (Additional file 5B). It contains a variable dictionary for Additional file 5B.

**Additional file 7: A** Across-state differences for WISN values for facilities in ABCE surveys for PHC HRH cadres. Figures for non-parametric statistical comparisons among states for PHC-nurses and PHC-doctors. **B** Across-state differences for WISN values for facilities in ABCE surveys for CHC HRH cadres. Figures for non-parametric statistical comparisons among states for CHC-nurses, CHC-GDMOs, CHC-physicians, CHC-surgeons, CHC-OBGYNs, and CHC-paediatricians.

**Additional file 8:** Maps for overall WISN differences for doctors and nurses at primary and community health centres (PHCs and CHCs).

**Additional file 9:** Maps for sanctioning differences for doctors and nurses at primary and community health centres (PHCs and CHCs).

## Data Availability

The facility data sets analysed in the current study have been previously reported under the Access, Bottlenecks, Costs, and Equity (ABCE) Project for Madhya Pradesh, Gujarat, Odisha, Tamil Nadu, Andhra Pradesh and Telangana. Data is available at the Global Health Data Exchange (GHDx) (http://ghdx.healthdata.org/series/access-bottlenecks-costs-and-equity-abce-project) from the Institute for Health Metrics and Evaluation (IHME). The Rural Health Statistics (RHS) 2019 data is available at (https://nrhm-mis.nic.in/Pages/RHS2019.aspx?RootFolder=%2FRURAL%20HEALTH%20STATISTICS%2F%28A%29%20RHS%20%2D%202019&FolderCTID=0x01200057278FD1EC909F429B03E86C7A7C3F31&View={473F70C6-7A85-47C5-AB5C-B2AD255F29B2}) from the Ministry of Health and Family Welfare, Government of India. All data sets generated during this study are included in this article and its Additional files.

## Abbreviations

WISN: Workforce Indicators of Staffing Need
RHS: Rural Health Statistics
ABCE: Access, Bottlenecks, Costs, Equity
NHPI: National Health Policy of India
NRHM: National Rural Health Mission
IPHS: Indian Public Health Standards
PHC: Primary Health Centre
CHC: Community Health Centre
GDMO: General Duties Medical Officer
OBGYN: Obstetrician and Gynaecologist
MP: Madhya Pradesh
GJ: Gujarat
OD: Odisha
TN: Tamil Nadu
AP&TG: Andhra Pradesh and Telangana
