# Supplementary figures and images for: Workforce problems at rural public health-centres in India: a WISN retrospective analysis and national-level modelling study
Source: Hum Resour Health. 2022 Jan 28;19(Suppl 1):147. doi: 10.1186/s12960-021-00687-9 (PMC8796332; doi:10.1186/s12960-021-00687-9)

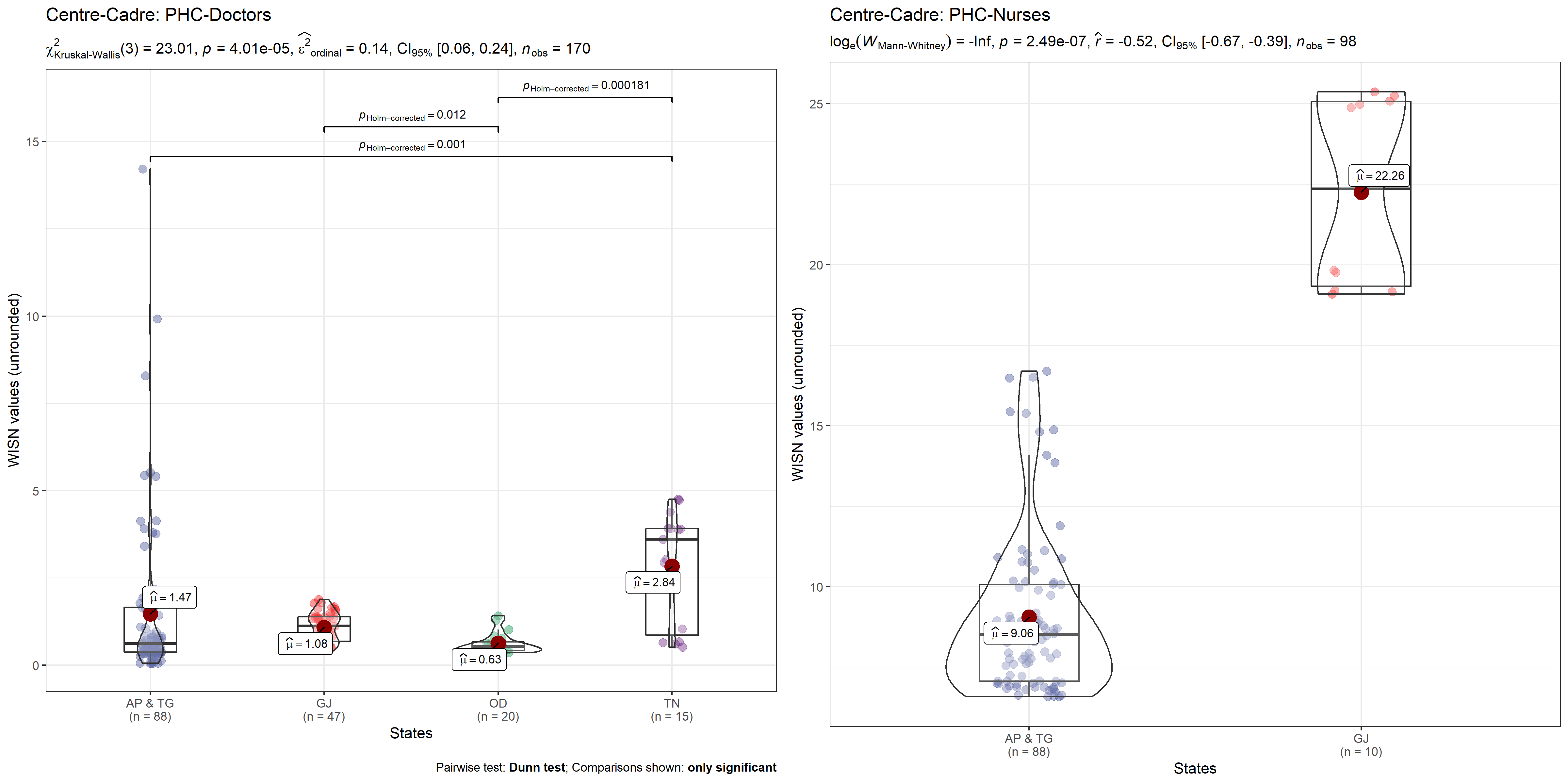

Supplement: Supplementary file 7 — Additional file 7: A Across-state differences for WISN values for facilities in ABCE surveys for PHC HRH cadres. Figures for non-parametric statistical comparisons among states for PHC-nurses and PHC-doctors. B Across-state differences for WISN values for facilities in ABCE surveys for CHC HRH cadres. Figures for non-parametric statistical comparisons among states for CHC-nurses, CHC-GDMOs, CHC-physicians, CHC-surgeons, CHC-OBGYNs, and CHC-paediatricians. [file 12960_2021_687_MOESM7_ESM.zip › WISN_Additional_File_7AR0.png]

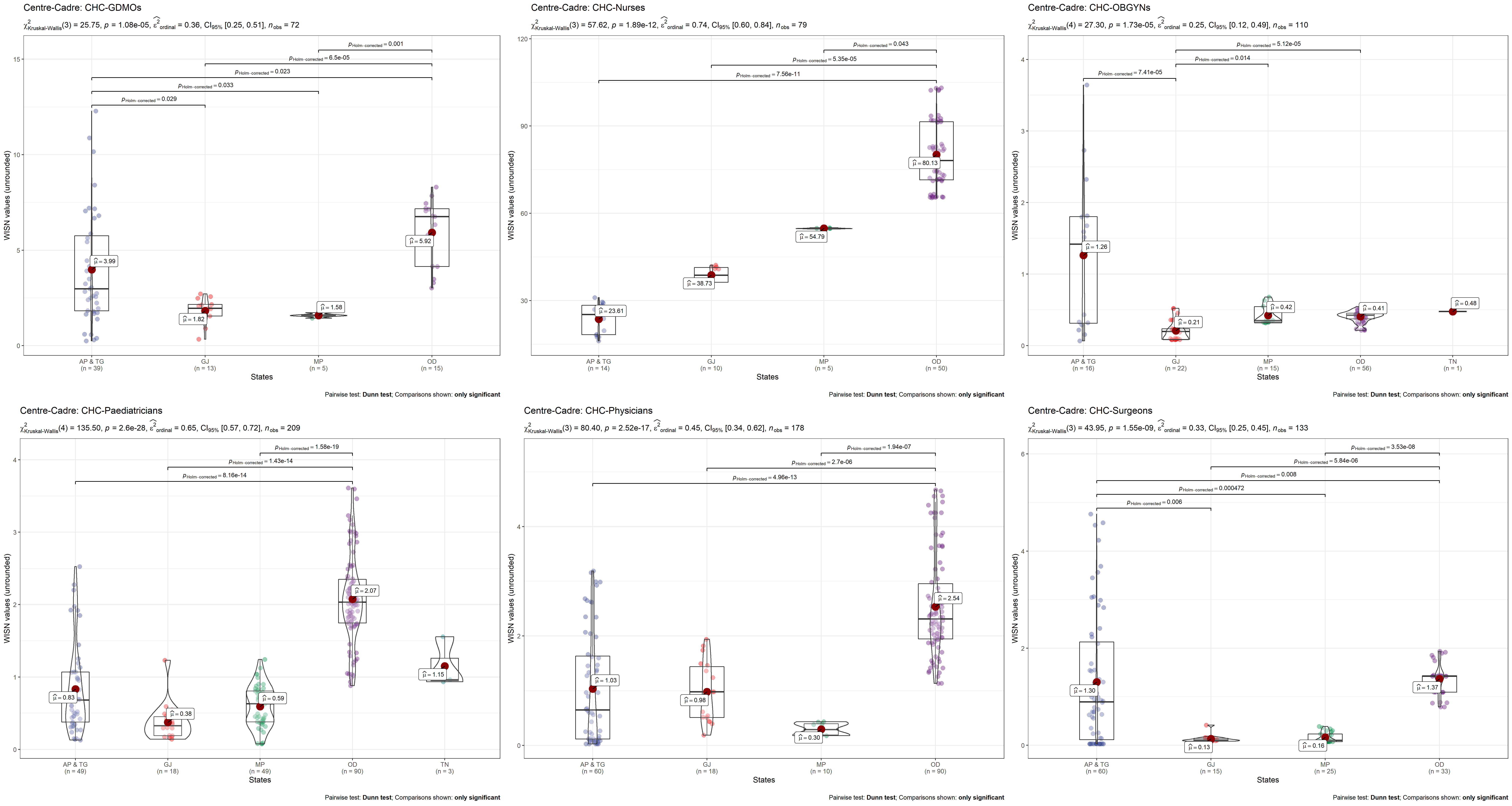

Supplement: Supplementary file 7 — Additional file 7: A Across-state differences for WISN values for facilities in ABCE surveys for PHC HRH cadres. Figures for non-parametric statistical comparisons among states for PHC-nurses and PHC-doctors. B Across-state differences for WISN values for facilities in ABCE surveys for CHC HRH cadres. Figures for non-parametric statistical comparisons among states for CHC-nurses, CHC-GDMOs, CHC-physicians, CHC-surgeons, CHC-OBGYNs, and CHC-paediatricians. [file 12960_2021_687_MOESM7_ESM.zip › WISN_Additional_File_7BR0.png]

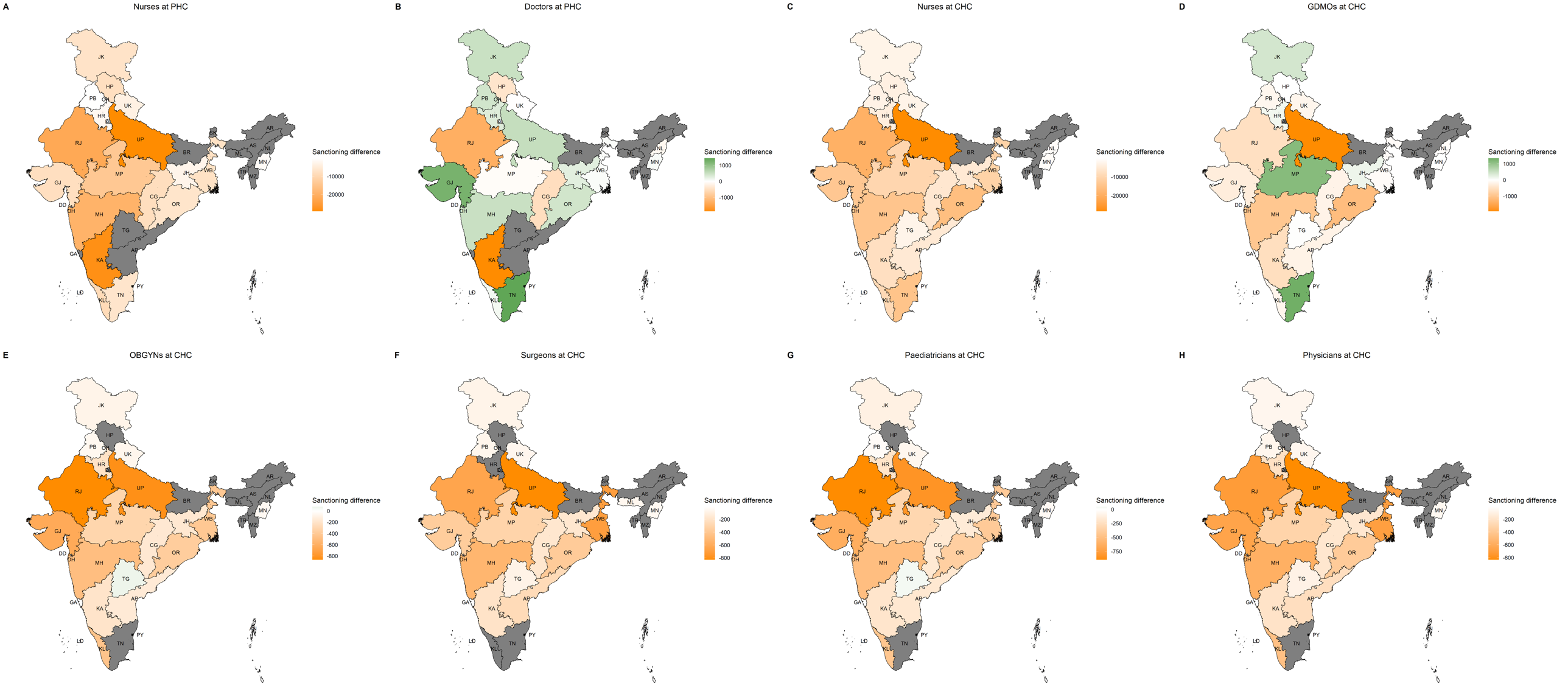

Supplement: Supplementary file 8 — Additional file 8: Maps for overall WISN differences for doctors and nurses at primary and community health centres (PHCs and CHCs). [file 12960_2021_687_MOESM8_ESM.png]

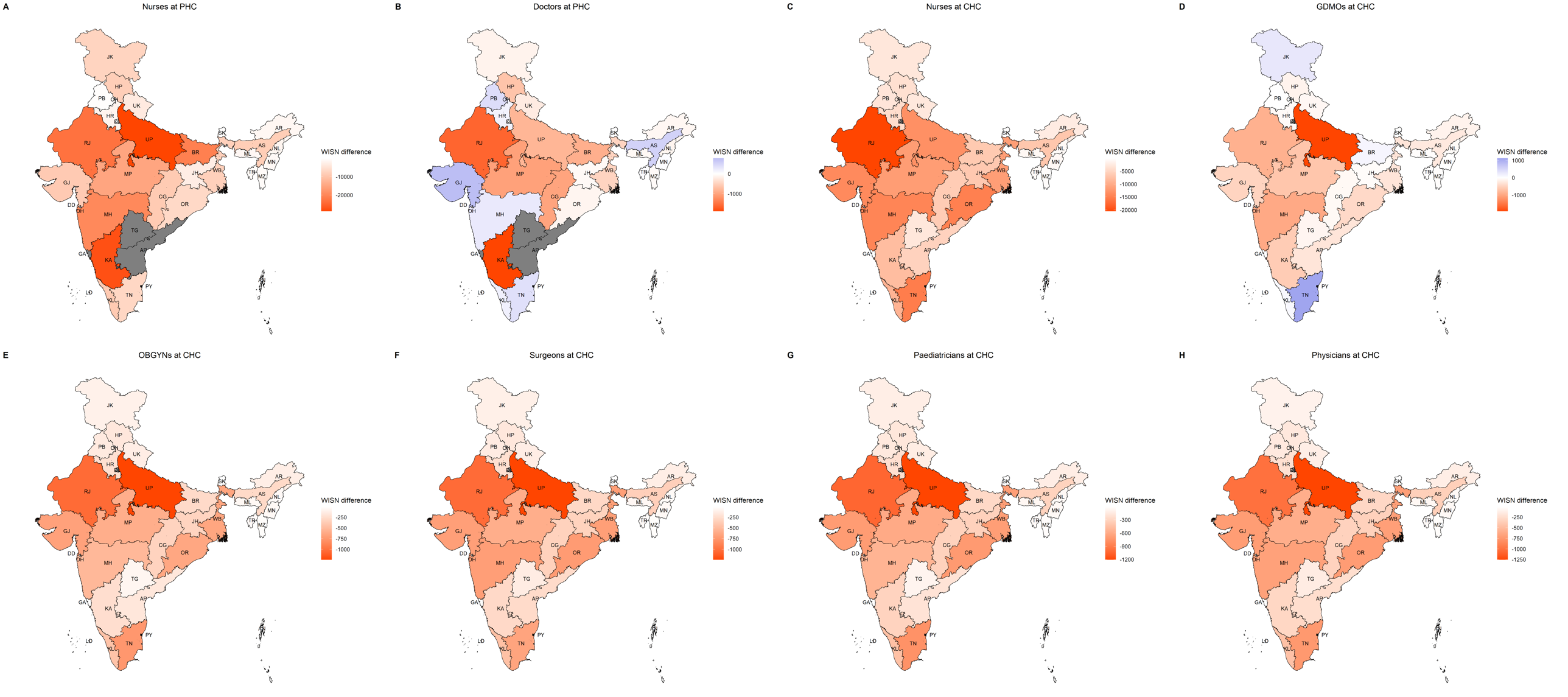

Supplement: Supplementary file 9 — Additional file 9: Maps for sanctioning differences for doctors and nurses at primary and community health centres (PHCs and CHCs). [file 12960_2021_687_MOESM9_ESM.png]
